# Exploring the Effect of Esomeprazole on Gastric and Duodenal Fluid Volumes and Absorption of Ritonavir

**DOI:** 10.3390/pharmaceutics12070670

**Published:** 2020-07-17

**Authors:** Tom de Waal, Jari Rubbens, Michael Grimm, Vincent Vandecaveye, Jan Tack, Werner Weitschies, Joachim Brouwers, Patrick Augustijns

**Affiliations:** 1 Drug Delivery and Disposition, KU Leuven, Gasthuisberg O&N II, Herestraat 49—Box 921, 3000 Leuven, Belgium; tom.dewaal@kuleuven.be (T.d.W.); jari.rubbens@just.fgov.be (J.R.); joachim.brouwers@kuleuven.be (J.B.); 2 Center of Drug Absorption and Transport, Institute of Pharmacy, University of Greifswald, 17489 Greifswald, Germany; michael.grimm@uni-greifswald.de (M.G.); werner.weitschies@uni-greifswald.de (W.W.); 3 Radiology Department, University Hospitals Leuven, 3000 Leuven, Belgium; vincent.vandecaveye@uzleuven.be; 4 Translational Research Center for Gastrointestinal Disorders, TARGID, KU Leuven, 3000 Leuven, Belgium; jan.tack@kuleuven.be

**Keywords:** MRI, PPI effect, ritonavir, gastric fluid volume, intestinal fluid volume, drug absorption

## Abstract

Proton-pump inhibitors (PPIs), frequently prescribed to lower gastric acid secretion, often exert an effect on the absorption of co-medicated drug products. A previous study showed decreased plasma levels of the lipophilic drug ritonavir after co-administration with the PPI Nexium (40 mg esomeprazole), even though duodenal concentrations were not affected. The present study explored if a PPI-induced decrease in gastrointestinal (GI) fluid volume might contribute to the reduced absorption of ritonavir. In an exploratory cross-over study, five volunteers were given a Norvir tablet (100 mg ritonavir) orally, once without PPI pre-treatment and once after a three-day pre-treatment with the PPI esomeprazole. Blood samples were collected for eight hours to assess ritonavir absorption and magnetic resonance imaging (MRI) was used to determine the gastric and duodenal fluid volumes during the first three hours after administration of the tablet. The results confirmed that PPI intake reduced ritonavir plasma concentrations by 40%. The gastric residual volume and gastric fluid volume decreased by 41% and 44% respectively, while the duodenal fluid volume was reduced by 33%. These data suggest that the PPI esomeprazole lowers the available fluid volume for dissolution, which may limit the amount of ritonavir that can be absorbed. Although additional factors may play a role, the effect of PPI intake on the GI fluid volume should be considered when simulating the absorption of poorly soluble drugs like ritonavir in real-life conditions.

## 1. Introduction

Since their introduction three decades ago, proton-pump inhibitors (PPI) have taken over as the leading drug class in the treatment and prevention of various gastric acid related diseases (e.g., gastroesophageal reflux disease (GERD), peptic ulcer bleeding and non-steroidal anti-inflammatory drug (NSAID) induced ulcers) [1]. Their mechanism of action is related to the covalent binding of the PPI to the H^+^/K^+^ ATPase in the acid secreting parietal cells. By targeting the final and universal step in the complex and highly regulated acid secreting pathway, they outperform their predecessors in terms of efficacy and safety. However, as PPIs are so frequently (over)prescribed, concern has recently been risen regarding the adverse effects of prolonged PPI treatment. Adverse effects include *C. difficile*-associated diarrhoea and increased risk of acute and chronic kidney disease, and myocardial infarction [2]. In addition, malabsorption of calcium, iron, magnesium and vitamin B12 are reported as their absorption depends on the acidic gastric fluid [3].

With respect to the absorption of orally administered drugs, the change in gastric pH due to PPI treatment may affect poorly soluble basic drug compounds that rely on the gastric acidity to dissolve and remain in a supersaturated state upon entering the small intestine creating the driving force for absorption [4]. Drug-drug interactions associated with PPIs are not only ascribed to the effect on gastric pH, but also to metabolic enzyme competition (i.e., CYP2C19 and CYP3A4) and some non-specified effects [5,6,7,8]. Another effect of PPI treatment is that a reduced gastric fluid secretion lowers the fluid volume available in the stomach, which may have serious consequences considering the large fraction of lipophilic and poorly water soluble drug candidates that need sufficient fluid to dissolve and be absorbed [9,10,11]. As PPIs remain one of the most frequently prescribed drugs, it is essential to understand the effects they pose to the absorption of co-medicated drugs to ensure reliable and safe therapies for patients [12]. In particular, a better insight into the mechanisms underlying drug-PPI interactions is important to optimize the preclinical evaluation of oral drug products. Only when the relevant mechanisms are simulated in, for instance, in vitro dissolution testing and in silico absorption modelling, the robustness of oral drug products to PPI-induced changes in the gastrointestinal environment can be adequately evaluated.

In a recent study in healthy human volunteers, Van Den Abeele et al. assessed the effect of the PPI esomeprazole on the luminal and systemic concentrations of the BCS class IV drug ritonavir, an HIV protease inhibitor often used as a booster with other HIV protease inhibitors [13]. Ritonavir is a lipophilic (Log *P* 4.3) weak base (p*Ka* 2.6) with pH-dependent solubility, formulated as an amorphous solid dispersion (Norvir 100 mg) [14]. In normal fasted state conditions, the solubility of ritonavir is, therefore, much higher in the acidic stomach than in the small intestine (e.g., 77.9 µg/mL in fasted state simulated gastric fluid at pH 1.6 vs. 11.5 µg/mL in fasted state simulated intestinal fluid at pH 6.5) [15]. It can be expected that the reduced acidity of the stomach upon PPI treatment will reduce the gastric solubility of ritonavir and substantially affect its dissolution behaviour. Indeed, the study of Van Den Abeele et al. showed that the solid fraction of ritonavir in the stomach (undissolved and/or precipitated) was elevated when the PPI esomeprazole was co-administered. In the small intestine, however, dissolved concentrations did not differ between the control vs. PPI condition. In both cases, duodenal ritonavir concentrations were supersaturated (i.e., the dissolved concentration exceeded the intrinsic solubility), clearly indicating the enabling effect of the amorphous solid dispersion formulation. Despite the fact that duodenal concentrations were unaffected, PPI treatment did reduce the systemic exposure to ritonavir in most of the subjects. The most obvious explanation for the decrease in systemic exposure would be that a decreased solubility of ritonavir in a PPI-treated stomach results in lower ritonavir concentrations in the duodenum (less supersaturation) and, thus, a reduced driving force for absorption. However, this is contradicted by the similar dissolved concentrations in the small intestinal lumen. An alternative hypothesis is that the available fluid volume in the gastrointestinal tract is decreased upon PPI treatment, which would imply that similar dissolved concentrations do not reflect the total amount of the dose that is in solution and available for absorption. Although the effect of PPIs on the fluid volume in the stomach was addressed in the past, the extent and impact on the absorption of co-medicated drugs remain unclear. In this respect, it is important to note that data regarding the effect of PPIs on the small intestinal fluid volume are non-existing, even though changes in this volume may have a very large impact as it is considered to be the main site of drug absorption. Considering amorphous solid dispersions, sufficient fluid must be present to rapidly dissolve solid material or maintain supersaturation upon transfer of dissolved drugs from the stomach to the small intestine.

In the present study, an exploratory cross-over clinical trial was performed similar to the one described above [13]. Systemic concentrations of ritonavir were determined upon oral administration of a Norvir tablet to healthy volunteers, whether or not pre-treated with the PPI esomeprazole. Instead of intraluminal sampling, however, the gastric and duodenal fluid volumes were assessed using magnetic resonance imaging (MRI). MRI has previously been used as a reliable non-invasive method to assess gastric, small intestinal and even large intestinal fluid volumes, and is capable to differentiate between small volume changes [16,17,18]. This approach allowed us to explore (i) the effect of the PPI esomeprazole on gastric and, for the first time, duodenal fluid volumes in humans, and (ii) whether such an effect may contribute to the interaction between esomeprazole and the poorly soluble drug ritonavir. The outcome of this study may guide further research to better simulate PPI effects on oral drug products.

## 2. Material and Methods

### 2.1. Materials

#### 2.1.1. Chemicals

Acetonitrile (ACN) and methanol (MeOH) (LC-MS grade) were purchased from Biosolve (Valkenswaard, the Netherlands). Methanol (HPLC grade) and dimethylsulfoxide (DMSO, 99.9%, for spectroscopy) were ordered from Acros Organics (Geel, Belgium). Ethyl acetate (EtOAc, 99.9%) were purchased from VWR (Haasrode, Belgium), while Chem-Lab (Zedelgem, Belgium) supplied hexane (C_6_H_14_, >99%, p.a.). Hanks’ Balanced Salt Solution without phenol red (HBSS) was ordered from Lonza (Verviers, Belgium). Merck (Darmstadt, Germany) supplied sodium hydroxide (NaOH, pellets, p.a.) and disodiumtetraborate decahydrate (Na_2_B_4_O_7_.10H_2_O, borax, 99–103%). Ritonavir (RTV) powder was purchased from Sigma-Aldrich (St. Louis, MO, USA.) for analytical purposes. Lopinavir (LPV) powder was donated by Hetero Drugs Ltd. (Hyderabad, India). Water was purified using a Maxima System (Elga Ltd., High Wycombe Bucks, UK).

#### 2.1.2. Study Medication

Norvir (100 mg ritonavir; Abbvie, North Chicago, IL, USA) and Nexium (40 mg esomeprazole; AstraZeneca, London, UK) tablets were ordered via the hospital pharmacy of the University Hospitals Leuven (UZ Leuven, Leuven, Belgium).

### 2.2. Methods

#### 2.2.1. Clinical Trial Design 

In this exploratory clinical trial, five healthy volunteers (three males and two females, aged between 19 and 22 years) participated in a two-arm crossover study (washout period: at least one week) comprising the following test conditions:

- Control condition: Oral intake of one Norvir tablet (100 mg ritonavir) with 240 mL of tap water after a fasting period of 12 h.
- PPI condition: Oral intake of one Norvir tablet (100 mg ritonavir) with 240 mL of tap water after a fasting period of 12 h preceded by a once-daily dose of the PPI esomeprazole (40 mg, i.e., one Nexium tablet) for three days (including the morning of the trial).

Possible volunteer candidates (aged 18–35 years) were excluded from the trial in the case of disease, chronic use of medication (birth control excluded), gastrointestinal pathology or prior surgery, hepatitis B/C and/or HIV infection, and (possible) pregnancy. The clinical trial was approved by the competent authority in Belgium (Federal Agency for Medicines and Health Products, EudraCT reference number: 2016-002700-78) and the Ethics Committee Research UZ/KU Leuven (S59578—amended), and was performed in accordance with the rules in the Declaration of Helsinki.

#### 2.2.2. Clinical Trial Protocol

All volunteers provided written informed consent before participating in the trial. Each trial day, the participant came to the radiology department at the University Hospitals Leuven after an overnight fast (at least 12 h). Water was allowed until the night before the trial except for the volume needed to swallow the PPI tablet. For the PPI condition, participants were pre-treated with a once-daily dose of Nexium (just before breakfast) starting two days prior to the trial day. On the morning of the trial, the final Nexium tablet was taken 2 h prior to the arrival time at the hospital, with as little water as possible to swallow the tablet. A daily dose of 40 mg esomeprazole for three days has shown to elevate and sustain the gastric and duodenal pH to values around 7.1 and 7.5, respectively [13], indicative of the acid-inhibitory effect [19,20]. A venous catheter was placed in the subject’s arm and a blank blood sample (5 mL) was collected in heparinized tubes (BD Vacutainer systems, Plymouth, UK). After administration of the Norvir tablet with 240 mL of tap water, blood samples (up to 5 mL) were collected to determine the ritonavir plasma concentration over an 8-h period: after 30, 55, 85, 115, 145, 175, 210, 240, 270, 300, 330, 360, 420, 480 min. In addition, subjects were placed in a Philips 1.5 T MRI System (Philips, Best, The Netherlands) to assess the gastric and duodenal fluid volume, once before administration of the study medication and afterwards at predetermined time points, i.e., 20, 40, 60, 90, 120, 150 and 180 min.

#### 2.2.3. Gastric and Duodenal Fluid Volume via MRI

Upon entering the MRI system, a rectangular dStream FlexCoverage Anterior coil (Ingenia 1.5 tesla, Philips, Best, The Netherlands) was placed on the torso of the subjects. A scouting sequence was used in the coronal and transversal plane to allow correct sequence positioning. At each time point, three standardized sequences were used to acquire images; sequence parameters are depicted in Table 1. Images were taken during exhalation breath hold.

**Figure 1:**
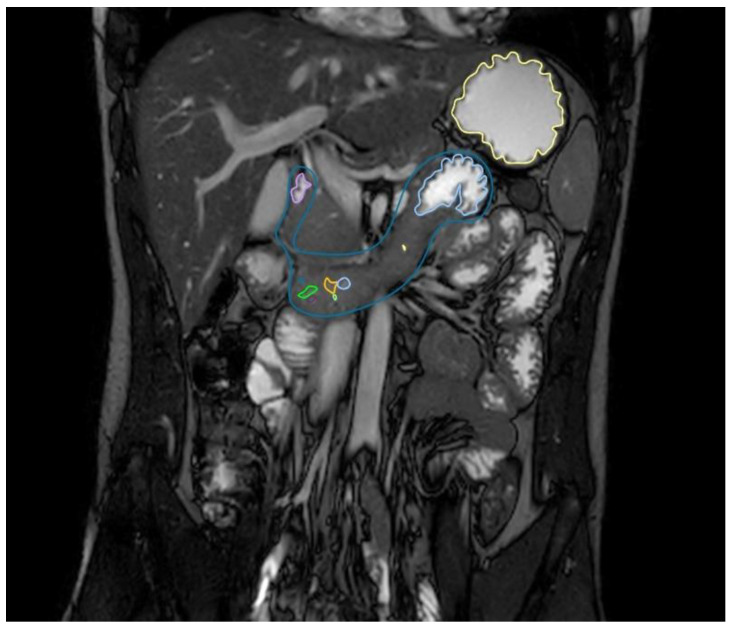
MRI TrueFISP sequence image in the coronal plane; the stomach (yellow outline) and duodenum (dark-blue outline) with the fluid pockets outlined in different colours.

Images were analysed using Horos™ (Version 3.3.6, Horos project), a free open source medical images viewer. Gastric and duodenal fluid volumes reported in this study were acquired with sequence 1. Although gastric fluid volumes determined with sequences 2 and 3 were similar, sequence 1 was chosen because (i) no artefacts were present (in contrast to sequence 2 where bands in the stomach were occasional on the outer border of the field of view) and (ii) both the stomach and duodenum were completely visible (in contrast to sequence 3 where the duodenum of some volunteers was not visible). Images made with sequence 2 yielded a superior detail of the small intestine; however, due to the ultra-rapid acquisition, both the vena cava and aorta have a similar contrast as the fluid in the intestines. This made the sequence unsuitable for fluid determination as the duodenum passes these blood vessels which would interfere with the outlining of the fluid present in the duodenum. Sequence 2 was therefore used as a localizer for the duodenum (i.e., determining the location of the duodenojejunal flexure (Figure 1).

As free water lights up in a T2 weighted image, a trained operator semi-automatically traced all the bright spots in each slice for the stomach and duodenum, according to a published procedure [21]. To differentiate between free water and other contents in the respective organs, a cut-off value (lower threshold) of 30% of the brightest voxel in either the stomach or the duodenum was applied. This cut-off value must be regarded as a rule of thumb and manual correction was made to account for bright organs like the gall bladder and obvious acquisition errors (e.g., illogical dark spots in the middle of a fluid filled stomach). Subsequently, a 3D image of the traced fluid was rendered and the fluid volume was calculated by adding up the fluid volumes of each slide (multiplying the surface area of the traced region of interest with the slice thickness).

#### 2.2.4. Ritonavir Systemic Drug Exposure by LC-MS/MS

##### Sample Preparation

Blood samples were stored on ice during the remainder of the study day, before centrifugation at 2880× *g*, for 10 min at 4 °C (Centrifuge 5804R, Eppendorf, Hamburg, Germany). Plasma was collected and stored at −20 °C pending analysis. For analysis, a liquid-liquid extraction was performed in which 50 µL of plasma was added to 450 µL HBSS and vortexed for a few seconds. Lopinavir was used as internal standard: 100 µL of a 200 nM stock solution in 25% *v*/*v* DMSO-water was added to the mixture. Before extraction, the pH was adjusted by adding 500 µL of 40 mM borax buffer (pH = 9), followed by 5 mL of the extraction medium hexane—ethyl acetate (50:50, *v*/*v*) and shaken for 1 min. The mixture was centrifugated at 2880× *g*, for 5 min at 4 °C and the organic layer was transferred to a new test tube and evaporated to dryness under a gentle airflow. The residue was redissolved in 1 mL of methanol and vortexed for 30 s before another evaporation cycle. The resulting residue was reconstituted in 200 µL methanol—water (50:50, *v*/*v*) awaiting analysis.

##### LC—MS/MS Analysis

For the analysis, a Waters ACQUITY UHPLC H-Class system consisting of a quaternary pump was used combined with a Waters Xevo TQ-S micro mass spectrometer (Waters, Milford, MA, USA). Separation took place on a Kinetex^®^ column (2.6 µm XB-C18 100 A, 50 × 2.1 mm, Phenomenex, Utrecht, the Netherlands) using isocratic elution with methanol—0.1% formic acid (65:35, *v*/*v*) followed by a wash gradient and re-equilibration (Table 2). Column temperature was set at 35 °C and samples were kept in the autosampler at 15 °C. After injecting 1 µL of sample, ritonavir and the internal standard lopinavir eluted at 1.79 min and 2.60 min, respectively. Peaks were detected with MS/MS, using electrospray ionization (ESI) as ion source. System parameters in positive ion mode were selected after tuning of ritonavir via direct infusion: capillary voltage 1.65 kV, cone voltage 25 V, desolvation gas (N_2_) flow 700 L/h and temperature 500 °C, and cone gas (N_2_) flow 400 L/h. Fragmentation was induced by collision with argon. The system was operated in multi-reaction monitoring (MRM) mode with the mass transitions according to Table 3.

The method was validated according to ICH guideline M10 on bioanalytical method validation and passed on selectivity, linearity and range (1 nM–4000 nM), intra- and interday accuracy (bias < 10%) and precision (relative standard deviation < 5%) tested on 4 QC levels (5 nM, 100 nM, 1000 nM and 4000 nM), recovery (95%) and freeze-thaw stability (100%). In addition, no matrix effect or degradation was observed during the experiments. Carryover was observed when higher concentrations were injected but was mitigated by blank injections. QC samples were included in each run to show method suitability.

#### 2.2.5. Data Presentation

The explorative nature of the study did not allow for a detailed statistical analysis. Results are presented as median (range) unless otherwise specified. Wherever appropriate, individual profiles are given to further clarify the observations made in this study.

## 3. Results

### 3.1. Systemic Disposition of Ritonavir

**Figure 2:**
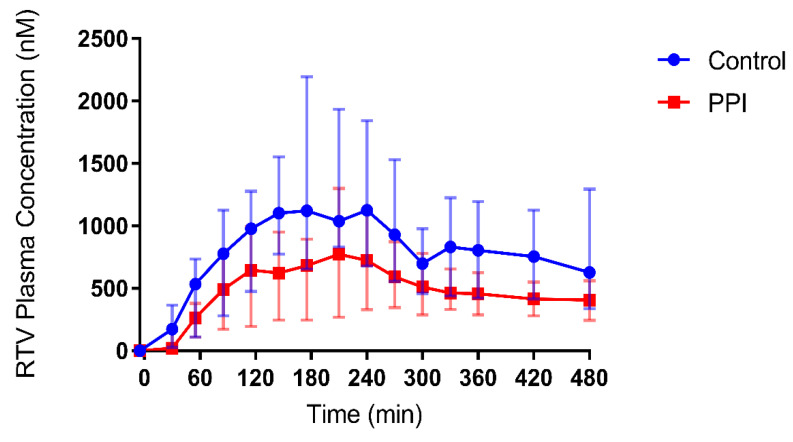
Median plasma concentration-time profiles for ritonavir (RTV) following oral intake of a Norvir tablet in control condition (blue dots) vs. PPI condition (red squares). The error bars depict range (n = 4).

**Figure 3:**
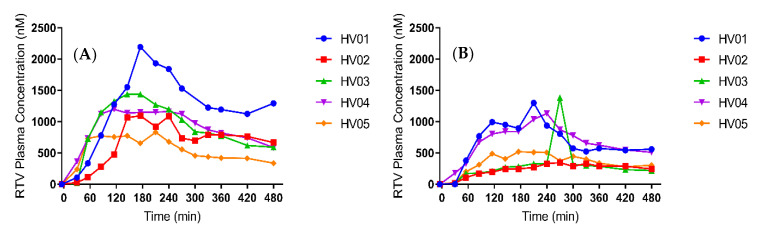
Plasma concentration-time profiles for ritonavir (RTV) in five healthy volunteers (HV) after oral intake of a Norvir tablet in (**A**) control condition and (**B**) PPI condition.

To confirm the effect of pre-treatment with the PPI esomeprazole on the absorption and systemic disposition of ritonavir, its plasma concentrations were determined after intake of a Norvir tablet. Figure 2 and Figure 3 depict the median and individual concentration-time profiles, respectively. A summary of the pharmacokinetic parameters is provided in Table 4. In one healthy volunteer (HV03), an aberrant ritonavir concentration was observed at 270 min after drug intake in the PPI condition, as it deviated more than three-fold from both the previous and the next time point (Figure 3b, HV03). Although this concentration was confirmed by re-analysis, contamination of the sample could not be ruled out. Therefore, the plasma concentration-time profiles from HV03 were excluded from calculation of the median C_max,_ t_max_ and AUC_0-480min_ in both control and PPI conditions, and from the median profiles depicted in Figure 2. 

**Figure 4:**
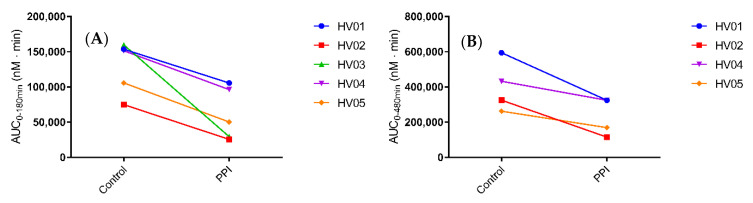
Systemic exposure to ritonavir in individual healthy volunteers (HV) after oral intake of a Norvir tablet in control vs. PPI condition, expressed as the area under the plasma concentration-time curve (AUC) (**A**) during the first 180 min (n = 5) and (**B**) during the full sampling period (480 min) period (n = 4).

Median C_max_ decreased from 1129 nM (range 833.0 to 2194 nM) in the control condition to 826.6 nM (329.6 to 1302 nM) in the PPI condition. In addition, median t_max_ slightly delayed from 193 min (range 175–240 min) to 225 min (175–270 min). Despite a relatively large intersubject variability, both the median and individual plasma concentration-time profiles (Figure 2 and Figure 3, respectively) show a clear decrease in ritonavir absorption in the PPI condition compared to control. In each of the five healthy volunteers, the extent of systemic drug exposure during the course of the MRI investigation (i.e., AUC_0-180min_) decreased upon PPI pre-treatment (Figure 4a), with a median relative decrease of 52.4% (range 31.3–81.6%). Similar results were observed for the AUC_0-480min_ (median relative decrease 40.5%, range 24.9–64.5%) (Figure 4b). Overall, the systemic disposition data suggest that pre-treatment with the PPI esomeprazole substantially reduces the rate and extent of ritonavir absorption.

### 3.2. Gastric Fluid Volume

**Figure 5:**
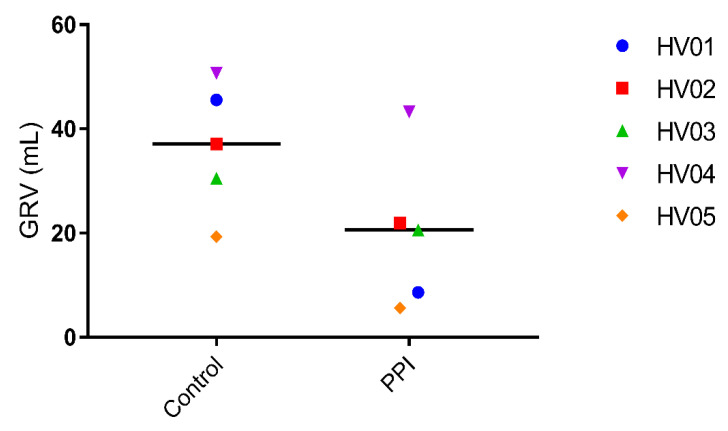
Gastric residual volume (GRV) in control (left) and PPI (right) condition, in five human volunteers (HV); the line depicts the median.

To explore a possible link between the observed PPI effect on ritonavir absorption and gastrointestinal fluid volumes, we first determined the gastric residual volume (GRV), i.e., prior to ritonavir intake. Compared to the control condition, PPI pre-treatment lowered the GRV in all volunteers, with a relative decrease ranging from 14.6% to 81.1% (Figure 5).

**Figure 6:**
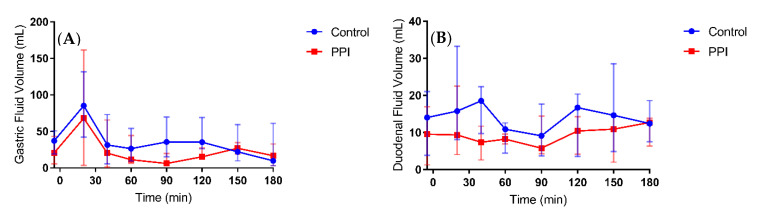
Median (**A**) gastric fluid volume and (**B**) duodenal fluid volume over time profiles following oral intake of a Norvir tablet with 240 mL water in control condition (blue dots) vs. PPI condition (red squares). The error bars depict range (n = 5).

**Figure 7:**
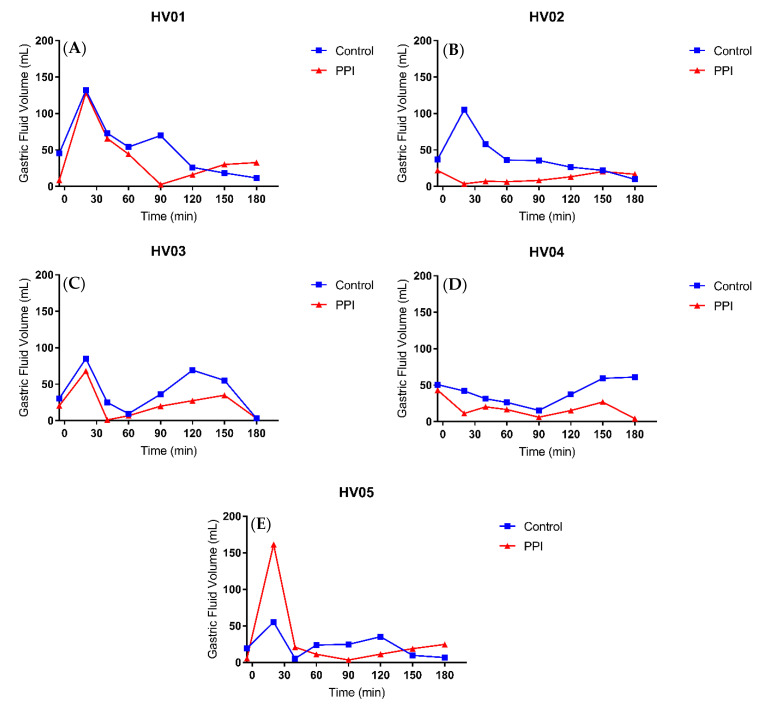
Individual gastric fluid volume over time profiles for (**A**) HV01, (**B**) HV02, (**C**) HV03, (**D**) HV04 and (**E**) HV05 following oral intake of a Norvir tablet with 240 mL water in control (blue squares) vs. PPI condition (red triangles).

**Figure 8:**
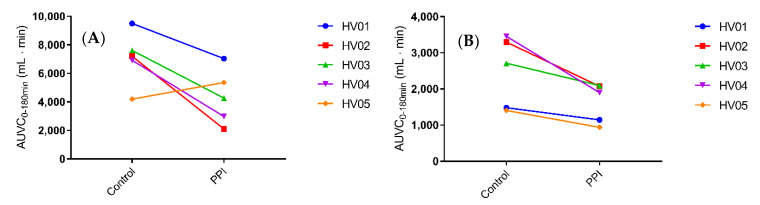
Total individual (**A**) gastric fluid volume and (**B**) duodenal fluid volume in five healthy volunteers (HV) following oral intake of a Norvir tablet in control condition vs. PPI condition, expressed as the area under the volume curve (AUVC) during the first 180 min.

After intake of the ritonavir tablet with 240 mL of water, the median gastric fluid volume over time profiles depict generally reduced fluid volumes in the PPI condition compared to control (Figure 6a). The gastric fluid volume dropped more when volunteers were pre-treated with PPI as compared to the control condition (Figure 7). The increase in gastric fluid volume that followed the initial emptying phase was also much more pronounced in the control condition. In general, the rate of gastric emptying seemed not to be impacted as the slope of the median gastric volume over time profile after the initial peak was similar in both fasted and PPI condition (Figure 6a). This indicates that the PPI effect on the gastric fluid volume is due to a reduced secretion of gastric fluid rather than a change in gastric emptying. The PPI-induced median relative decrease in total gastric fluid volume over the course of the study, as represented by the AUVC (area under the fluid volume curve, Figure 8a), amounted to 43.9% (range increase of 27.9 to a decrease of 70.8%). Except for HV05, the gastric fluid volume decreased for all volunteers (Figure 8a). In HV05, the observed increase in AUVC could be attributed to the rapid gastric emptying in the first 20 min after tablet and water ingestion in the control condition compared to a much slower emptying in the PPI condition (Figure 7, HV05). After the initial fluid emptying, the decrease in fluid volume due to PPI pre-treatment became also obvious in HV05.

### 3.3. Duodenal Fluid Volume

**Figure 9:**
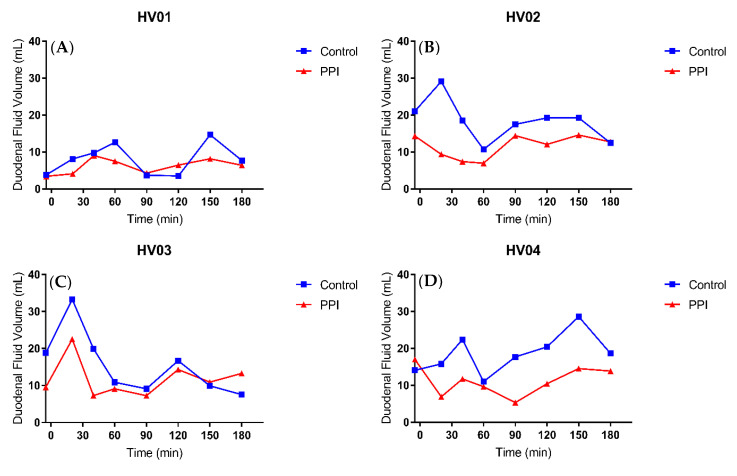

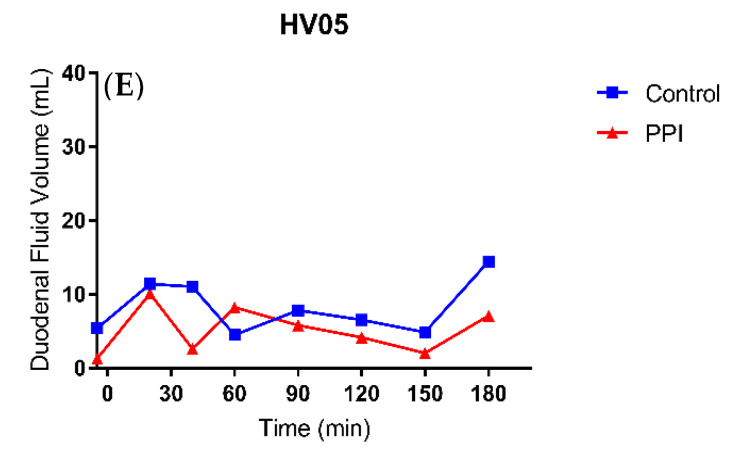
Individual duodenal fluid volume over time profiles for (**A**) HV01, (**B**) HV02, (**C**) HV03, (**D**) HV04 and (**E**) HV05 following oral intake of a Norvir tablet with 240 mL water in control (blue squares) vs. PPI condition (red triangles).

In comparison to the gastric fluid volume, the duodenal fluid volume appeared more stable, as can be seen in the median fluid volume over time profile (Figure 6b). Again, PPI pre-treatment resulted in a decreased fluid volume, both in offset (i.e., duodenal residual volume) and after tablet and water ingestion. The individual fluid volume over time profiles, depicted in Figure 9, provide a more detailed picture. The residual duodenal volume was decreased in three volunteers, similar in one volunteer and slightly increased in the last volunteer when control is compared to PPI. The course of the duodenal fluid volume did not follow the gastric fluid volume. For instance, the initial emptying of gastric fluid did not result in a distinct peak in the duodenal fluid volume. It is clear that duodenal fluid volume does not only depend on gastric emptying as the majority of fluid passes on quickly to the more distal parts of the intestine. Yet, there is an obvious effect of PPI pre-treatment on the overall duodenal fluid volume, as depicted in Figure 8B, with a median relative decrease in duodenal AUVC of 33.3% (range 22.7–45.1%).

## 4. Discussion

The effect of the PPI esomeprazole on the absorption of ritonavir has previously been investigated by Van Den Abeele et al., who first discovered that a decreased systemic exposure to ritonavir upon PPI pre-treatment could not be explained by a reduced driving force for absorption as the duodenal concentrations of ritonavir were unaffected [13]. By using the same study protocol but replacing gastrointestinal fluid aspiration with MRI to assess the intraluminal fluid volume, the present study aimed to further explore the underlying mechanism of the PPI effect on ritonavir absorption. Similar to the study of Van Den Abeele et al. [13], we demonstrated a clear effect of pre-treatment with the PPI esomeprazole on the absorption of ritonavir. When comparing the systemic exposure profiles of both studies, similar values were observed for the PPI-induced decrease in C_max_ (median 39.0% vs. 49.6%) and AUC_0–480min_ (median 40.5% vs. 39.5%). The delay in t_max_ differed between the studies (median 10% vs. 50%) but was highly variable in both. Due to the aberrant ritonavir plasma concentration observed after 270 min in HV03 (PPI condition), plasma concentrations from this volunteer were omitted from the calculations of C_max_, t_max_ and AUC_0-480min_. Irrespective of the aberrant point, however, the effect of PPI co-intake in HV03 was in line with the effect observed in the other volunteers, as can be seen in Figure 3. The similarities in PK parameters suggest that the differences in study protocol (i.e., the presence of nasal gastric tubes in the intestinal sampling study, and the positioning of the volunteers (alternating between supine and sitting up during the MRI study vs. semi-supine during the intestinal sampling study)) did not substantially affect ritonavir absorption. It should be noted that a systematic review by Béïque et al. [22], as well as a study by Morcos et al. [23], did not report an effect of another PPI (omeprazole) on plasma C_max_ or AUC of ritonavir. Chiu et al. [24] did observe a decreased ritonavir AUC when 40 mg omeprazole was dosed 2 h prior to an atazanavir/ritonavir tablet. In these studies, however, the PPI omeprazole was used, which is known to have a less pronounced effect on the gastric fluid volume compared to other PPIs (including esomeprazole, pantoprazole and rabeparzole) [11]. In addition, some studies did not use the ritonavir amorphous solid dispersion (Norvir), but a formulation that may not give rise to supersaturation of ritonavir in the small intestine, which may alter the absorption process and a possible interaction with PPIs.

Esomeprazole inhibits the H^+^/K^+^ ATPase of the parietal cells, thus reducing the gastric acid secretion. In addition, it has been shown that pre- and postprandial gastric fluid volume is decreased [4,11,25]. The fasted state GRV, observed in the present study, amounted to 37 ± 12 mL (mean ± SD), which is in agreement with the volumes published by Mudie et al. [18] (35 ± 7 mL) and by Schiller et al. [17] (45 ± 18 mL). Following pre-treatment with esomeprazole, the mean GRV reduced to 20 ± 15 mL. This highlights that the effect of the PPI is not only present after a meal where the gastric secretion is increased to facilitate digestion, but even in fasted state conditions when gastric secretion is thought to be very low [26]. Following intake of the ritonavir tablet with 240 mL water, the individual gastric fluid volume over time profiles (Figure 7) indicate that the PPI effect on the GRV remains visible once the administered water has been emptied from the stomach. It should be noted that the gastric fluid volume was highly variable, especially after 20 min. This reflects variation in the rate of gastric emptying of the administered water. In fasted state conditions, around 85% of a 240 mL dose of water is emptied within the first 30 min, but the rate is highly variable [16]. For this reason, the volumes observed after 20 min were similar to or lower than the GRV in some volunteers, while in others the ingested water was not yet completely emptied. The peak in gastric fluid volume due to the administration of water is therefore not or only minimally reflected in these profiles. Considering the excess of administered fluid in comparison to the GRV, however, it can be expected that the relative impact of PPI treatment on this peak volume is minimal. Overall, the data from the gastric fluid volume suggest that a daily dose of Nexium (40 mg esomeprazole) for three days is sufficient to reduce the gastric fluid secretion, thereby decreasing the available fluid for drug dissolution, which may affect drug absorption.

Small intestinal fluid volumes after ingestion of water in fasted state conditions have been determined previously by MRI, with Schiller et al. [17] reporting a mean small intestinal volume of 105 mL and Mudie et al. [18] reporting a mean peak volume of 92 mL decreasing to a steady volume around 77 mL. In both studies, there was a very large intersubject variability and a heterogenous distribution of the water pockets in the small intestine. In addition, Mudie et al. divided the intestine into quadrants to give an estimate of the location of fluid in the small intestine; they reported that most fluid resides in the jejunum and proximal ileum and that fluid in the duodenum is readily absorbed or passed through to the more distal parts of the intestine. Our data from the duodenal fluid volume are in line with the previous data, as there was less of a distinct peak in fluid volume, even though gastric emptying was rapid. In contrast to the gastric fluid volume, the duodenal fluid volume remained more or less steady (median 12.5 mL vs. 8.6 mL in control and PPI condition, respectively), although there was a large intrasubject variation in this volume. This study, however, is the first to report the duodenal fluid volumes after PPI treatment, which decreased in all volunteers with an average of 32%. In addition to the decreased gastric fluid, other factors must be considered to explain the drop in duodenal fluid volume. The elevated gastric pH reduces the secretion of secretin, resulting in diminished pancreatic and duodenal secretion as there is no need to neutralize the acidic gastric fluid [27]. It has also been observed in rats that PPIs may have a direct influence on the pancreas reducing the secretion [28]. Duodenal residual fluid volume was, in contrast to gastric residual volume, not decreased in all subjects (i.e., three out of five). It might be argued that this is due to the water ingested the morning of the PPI condition to swallow the esomeprazole tablet (2 h before the trial); this volume was not standardized but kept as low as possible and is likely to have varied between individuals. Although it cannot be ruled out that some of the ingested water was still present in the duodenum after 2 h, the remaining volume was probably very low, considering the rapid transfer of ingested water from the stomach through the duodenum to more distal parts of the intestine in fasted state conditions.

Even though MRI is used by multiple research groups to determine gastrointestinal fluid volumes, there are some limitations that must be addressed when determining small intestinal fluid volumes. In contrast to the stomach, which is clearly distinguishable and easy to outline via MRI, the small intestine is much more difficult to outline especially when low volumes are present. In addition, the small intestine is in constant motion, which implies the need to use fast acquisition during MRI by means of, for instance, a TrueFISP sequence (i.e., sequence 2 in the present study). Using TrueFISP, however, the aorta and vena cava interfere as they light up like fluids. For this reason, it was decided to use sequence 1 (turbo spin-echo) with a slower acquisition but without interference of the blood vessels, to determine gastric and duodenal fluid volumes. To accurately outline the duodenum and account for the intestinal movement, however, the TrueFISP (sequence 2) was used as a localizer for the duodenum and duodenojejunal flexure. Due to the inherent slicing (i.e., slice thickness) of the MRI, a slight under- or overestimation is easily made when the slice thickness is large compared to the intestinal distention. However, by considering these minor flaws (i.e., reduce slice thickness and interslice gap) and by standardization of the sequence protocol and data analysis, one can obtain accurate and comparable results in a non-invasive manner [21].

The present study confirmed that pre-treatment with the PPI esomeprazole substantially lowers the rate and extent of ritonavir absorption. By combining the PK study with MRI, the study further brought forward reduced gastric and duodenal fluid volumes as a viable mechanism behind the decreased exposure. The performance of amorphous solid dispersions, like Norvir, is highly depending on the composition and volume of the dissolution media [29]. Duodenal fluid may further dissolve solid material or maintain the supersaturated state originating from the stomach as long as the fluid volume is sufficient. Reduced fluid volumes upon PPI treatment may therefore imply that a lower fraction of ritonavir is in solution and available for absorption, despite similar concentrations in the duodenum.

It is important to note that the PPI-induced decrease in duodenal fluid volume is relatively limited as compared to the decrease in ritonavir plasma concentration (e.g., HV02 with an 65% drop in plasma AUC compared to just 37% drop in duodenal fluid AUVC). In addition, no direct correlation could be made between the gastric or duodenal fluid volume and the ritonavir plasma concentration in this small-scale study, raising the question if more factors are in play. Of course, one must take into account that, in this study, only duodenal volumes are reported, while drug absorption takes place along the entire small intestine. Fluid volumes in more distal parts (i.e., the jejunum and ileum) may also decrease with PPI treatment, possibly strengthening the overall effect. Another factor which must be considered is the first-pass metabolism of ritonavir, which may play an important role in the observed difference. Ritonavir is mainly metabolized in the liver by the CYP3A family and is, in itself, a potent inhibitor of CYP3A4; a smaller fraction is metabolized by CYP2D6. Studies have shown that the metabolism of ritonavir has at least one saturable process that is quickly saturated even after a single dose [30]. Following PPI-pretreatment, the absorption of ritonavir seems to be more gradual as can be observed by the flattened median profile compared to the control condition (Figure 2), and indicated by the lower and delayed plasma C_max_ in the present study. The more gradual exposure of ritonavir to the metabolic enzymes may reduce the saturation of the metabolic pathways leading to a more extensive metabolism, which could strengthen the PPI effect on the systemic exposure to ritonavir. It should be noted that the PPI esomeprazole is mainly metabolized by CYP2C19 and to a lesser extent by CYP3A4. Direct drug-drug interactions due to competition for CYP3A4 have been investigated, but no clinically relevant interactions have been reported [8]. Moreover, a competitive effect would lead to an increase in ritonavir exposure after PPI treatment (reduced elimination), while a decrease was observed.

Although the exploratory design of the present study does not allow formal conclusions, the observed median and individual data clearly suggest that intake of the PPI esomeprazole may reduce fluid volumes in not only the stomach but also the duodenum. A decrease in the fluid volume available for dissolution may contribute to the observed reduction in systemic exposure of the poorly soluble drug ritonavir upon PPI co-administration. The precise impact of the observed fluid volume changes, in combination with the PPI-induced drop in gastric pH, on the absorption of ritonavir will be further evaluated using in vitro dissolution testing in combination with physiologically-based pharmacokinetic (PBPK) modelling. PBPK modelling has previously been utilized to predict in vivo drug-drug interactions between weakly basic compounds and PPIs or other acid-reducing agents [31,32,33]. From a broader perspective, the results of this study should be taken into consideration when developing and testing new drug candidates especially for populations in which PPI use is most frequent, including elderly [12]. In vitro experiments tailored to account for fluid volume changes can give valuable information on the gastrointestinal behaviour of oral drug products that are frequently co-administered with PPIs or in populations where GI fluid volume may be impacted [4]. In addition, PBPK models can allow evaluating the sensitivity of drug absorption to relevant changes in gastrointestinal fluid volume [34,35].

## Figures and Tables

**Table 1:** MRI sequence parameters.

	Sequence 1	Sequence 2	Sequence 3
**Type**	T2-weighted turbo spin-echo	T2/T1 TrueFISP	T2-weighted turbo spin-echo
**Plane**	Coronal	Coronal	Axial
**Repetition time (ms)**	591.75	4.83	579.66
**Echo time (ms)**	120	2.41	120
**Acquisition matrix**	344 · 300	328 · 329	288 · 250
**Slice thickness (mm)**	4	4	4
**Interslice gap (mm)**	0.6	0	0.6
**Number of averages**	1	1	1
**Acquisition time (s)**	23.67	18.45	22.57

**Table 2:** LC parameters for the analysis of ritonavir in plasma samples.

Time (min)	Flow Rate (mL/min)	Methanol (%)	0.1% FA in Water (%)
**0**	0.500	65	35
**2.60**	0.500	65	35
**3.00**	0.500	95	5
**4.00**	0.500	95	5
**4.50**	0.500	65	35
**6.00**	0.500	65	35

**Table 3:** MS/MS parameters for the analysis of ritonavir in plasma samples.

Compound	Transition (*m*/*z*)	Collision Energy (V)	Function
**Ritonavir**	721.20 > 139.96	66	Quantification
	721.20 > 296.11	18	Identification
**Lopinavir**	629.35 > 155.15	49	Quantification

**Table 4:** Pharmacokinetic parameters of the ritonavir plasma concentration-time profiles following oral intake of a Norvir tablet in the control and PPI conditions. Median (range) with n = 4.

	Control	PPI
*C_max_* (nM)	1129 (833.0–2194)	826.6 (329.6–1302)
*t_max_* (min)	192.5 (175–240)	225 (175–270)
*AUC_0-480min_* (nmol·min/L)	3.8 · 10^5^ (2.6 · 10^5^–5.9 · 10^5^)	2.5 · 10^5^ (1.2 · 10^5^–3.2 · 10^5^)

## Abbreviations

AUC, Area under the plasma concentration-time curve; AUVC, Area under the fluid volume-time curve; CYP, Cytochrome P450; GI, Gastrointestinal; PPI, Proton-pump inhibitor.
